# Maintaining oxaliplatin therapy after hypersensitivity reactions: real-world experience with a desensitisation protocol

**DOI:** 10.1007/s10238-025-02035-w

**Published:** 2026-01-21

**Authors:** Eleftherios Christodoulis, Panagiotis J. Vlachostergios, Jacqueline Connell, Joseph Williams, Jurjees Hasan, Wasat Mansoor, Saifee Mullamitha, Richard A. Hubner, Michael Braun, Mark P. Saunders, Francisca Marti Marti, Angelos Angelakas, Tom Waddell, Nooreen Alam, Konstantinos Kamposioras

**Affiliations:** 1 Department of Medical Oncology, The Christie NHS Foundation Trust, Manchester, M20 4BX UK; 2 Division of Hematology & Medical Oncology, Weill Cornell Medicine, New York, NY 10065 USA; 3 Department of Medical Oncology, IASO Thessalias Hospital, Larissa, 41500 Greece; 4 Division of Pharmacy and Optometry, School of Health Sciences, The University of Manchester, Manchester, M13 9PT UK; 5 Department of Clinical Oncology, The Christie NHS Foundation Trust, Manchester, M20 4BX UK; 6 Division of Cancer Sciences, Faculty of Biology, Medicine and Health, The University of Manchester, Manchester, UK

## Abstract

Hypersensitivity reactions (HSRs) to oxaliplatin occur in 7–25% of patients and pose a significant clinical challenge, particularly for individuals who otherwise benefit from oxaliplatin-based therapy. Although evidence supporting its efficacy remains limited, drug desensitisation protocols (DDPs) involving stepwise dose escalation have been adopted in clinical practice. This study describes the experience of a tertiary cancer centre with oxaliplatin desensitisation, focusing on recurrence of HSRs and treatment completion rates. A retrospective observational study was conducted at a comprehensive cancer centre between October 2019 and January 2024. Clinicopathological characteristics and oncological outcomes were examined for patients with gastrointestinal malignancies who received oxaliplatin-based chemotherapy using a DDP. Sixty-six patients underwent oxaliplatin desensitisation (median age 60 years; 55% female). Most had colorectal cancer (CRC) (*n* = 35, 53%) or upper gastrointestinal malignancies (*n* = 26, 39%); 85% (*n* = 56) were treated with palliative intent. The median number of oxaliplatin cycles prior to the first HSR was six (range 1–26). CAPOX (53%) and FOLFOX (42%) were the most common regimens associated with HSRs. Patients received a median of three cycles within the DDP (range 1–19). Most (76%) remained on their original systemic anti-cancer therapy without premature treatment modification. Sixteen patients (24%) experienced a recurrent HSR; however, 55 patients (83%) successfully completed their intended treatment. Outcomes during the desensitisation period included: no evidence of disease in eight patients (seven treated in the adjuvant setting), treatment response in 26 patients, and disease progression in 32 patients. Oxaliplatin desensitisation is feasible and enables most patients to continue systemic therapy, with low rates of treatment discontinuation and acceptable oncological outcomes. This approach should be considered for eligible patients who experience oxaliplatin-related HSRs to maintain access to effective chemotherapy.

## Introduction

Oxaliplatin is a cytotoxic agent that acts by interacting with DNA to form intrastrand and interstrand DNA cross-links, affecting DNA pairing, replication, and gene transcription, leading to cell death [1]. Oxaliplatin-based chemotherapy regimens are the standard of care in neo-adjuvant, adjuvant, and palliative settings, for malignancies mainly of the gastrointestinal tract (gastric, oesophageal, pancreatic, and colorectal cancer) [2–4]. Hypersensitivity reactions (HSRs) to oxaliplatin have been well described as an adverse event, with its incidence reported to be between 7.1% and 25% [5–9], with some studies reporting even higher rates [10], although only 0.5–3% represent severe reactions [11,12]. HSRs can involve every organ system including the skin and mucosal membranes (flushing, erythema, pruritus, urticaria, rash, angioedema, throat tightness, tongue swelling), respiratory (nasal congestion, sneezing, wheezing, dyspnoea, cough, O2 desaturation, chest tightness), cardiovascular (tachycardia, chest pain, presyncope, syncope, hypertension, hypotension), gastrointestinal (nausea/vomiting, diarrhoea, abdominal pain, bloating, reflux), neuromuscular (numbness/weakness, back pain, headache, rigors, seizures, unusual taste, other pain), or present with constitutional symptoms (diaphoresis, chills and fever) [13]. These are mostly IgE-mediated reactions, leading to activation of basophils, but cytokine release, characterised by increased IL-6 levels and activation of other types of inflammatory cells such as macrophages, can also participate in some of these reactions such as fever, chills and pain [13,14]. Non-immediate HSRs have also been described such as antibody-mediated thrombocytopenia [15] and immune complex mediated syndromes manifesting with urticaria and proteinuria [16]. Repeat exposure to oxaliplatin can be life-threatening for patients who have previously presented with HSR [15].

Since the early days of oxaliplatin use, drug desensitisation protocols (DDPs) have been employed to circumvent HSRs [17]. These protocols involve delivering the target dose of a drug in incremental amounts. This approach was adopted after it was proven that premedication with steroids and antihistamines alone was not an efficient way of preventing these reactions [18,19]. DDPs modify the immune response of patients, generating temporary tolerance to the causative agent by raising the anaphylactic threshold, via well-established cellular pathways [20]. DDPs have been proven to be powerful tools that enable patients to continue an oxaliplatin-based regimen without interruption, which could hinder the benefits of antineoplastic treatment and have a negative impact on cancer outcomes [21].

However, there is insufficient evidence to evaluate safety, particularly with regard to the likelihood and severity of breakthrough reactions (BTRs), i.e. repeated HSRs to oxaliplatin in patients receiving it via a DDP. Similarly, there is limited evidence to assess the effectiveness of the protocol in terms of oncological outcomes. Although evidence is still evolving, initial and breakthrough HSRs have been associated with various clinical and immunological risk factors. Several large cohort and mechanistic studies have attempted to identify predictors of oxaliplatin-related HSRs. While initial HSRs to oxaliplatin are associated with cumulative dosing and patient history [22–24], breakthrough reactions during desensitisation protocols are less well studied. A more comprehensive understanding of the potential risk factors for BTRs is crucial to enable the safe and widespread implementation of DDPs.

The oxaliplatin-DDP has been used extensively at our institution since March 2018, with a standardised approach consistently followed. This retrospective report summarises our real-world experience of tolerability and oncological outcomes in this patient population.

## Methods

### Study design

This retrospective, single-cohort study examined patients who received oxaliplatin-based chemotherapy with a desensitisation protocol after experiencing a HSR between October 2019 and January 2024. Data were collected independently by two healthcare professionals and updated in March 2024.

The study included patients with primary cancer of any site who had received at least one cycle of oxaliplatin via the desensitisation protocol, in line with local policy for assessing and managing HSRs to oxaliplatin. Patient demographics, characteristics, and outcomes were retrospectively retrieved from electronic medical records. The primary endpoint of the study was the incidence of BTRs, as defined by Common Terminology Criteria for Adverse Events (CTCAE) version 3.0 ^26^.

The study was approved by the Quality Improvement and Audit Committee of The Christie Hospital (Reference Number 3916).

### Selection of patients for DDP

Patients who were eligible for the oxaliplatin desensitisation regimen had either experienced a grade 2 HSR during their initial oxaliplatin treatment, or a grade 1 or 2 HSR during rechallenge with oxaliplatin. Patients who experienced a grade 1 HSR during the initial oxaliplatin treatment could continue with a prolonged regimen, with the oxaliplatin administered over four hours alongside routine premedication (rechallenge protocol). However, if they experienced a further HSR with any subsequent infusion, they switched to the desensitisation protocol, unless the reaction was grade 3–4. In this case, they should not receive any further oxaliplatin treatment. The decision to implement a DDP following a grade 2 reaction was guided by the treating team for each patient. Patients who experienced a grade 3 or 4 reaction or prolonged/unresolved grade 2 symptoms should not be re-treated with oxaliplatin. Patients who experienced BTRs while on the desensitisation protocol should discontinue oxaliplatin treatment permanently.

Patients presenting with mild oxaliplatin-related HSRs that did not require the introduction of a DDP, and patients receiving a prolonged infusion of oxaliplatin using a rechallenge protocol, were excluded from the analysis.

### Desensitisation protocol

**Fig. 1:**
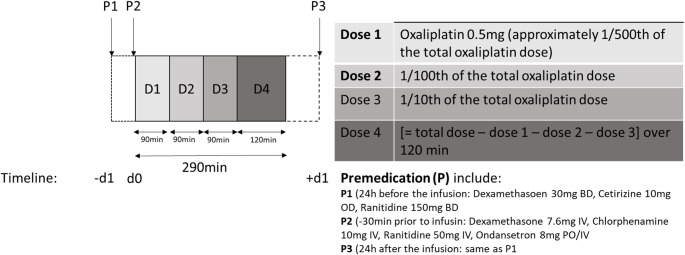
Oxaliplatin Desensitisation Protocol. This is a schematic diagram of the oxaliplatin desensitisation protocol that was used in our Institution for this study

The oxaliplatin desensitisation protocol was adapted from the carboplatin desensitisation protocol used at The Christie. This was done in collaboration with other UK tertiary cancer centres and in accordance with the available evidence at the time [26]. It included premedication with oral steroids (dexamethasone 4 mg BD) and oral antihistamines (cetirizine 10 mg and Famotidine 40 mg OD) starting the day before chemotherapy administration and continuing for a total of 3 days. Additionally, ondansetron (8 mg), dexamethasone (7.6 mg), chlorphenamine (10 mg) were given as bolus infusions and famotidine (40 mg orally) at least 30 min prior to oxaliplatin initiation. The oxaliplatin was administered in four separate escalating doses over 6.5 h. (dose 1 consisted of 1/500th of the total oxaliplatin dose infused over 90 min, dose 2 consisted of 1/100th of the total oxaliplatin dose infused over 90 min, dose 3 consisted of 1/10th of the total oxaliplatin dose infused over 90 min, and dose 4 consisted of the remaining oxaliplatin dose infused over 120 min). (Fig. 1).

It should be noted that oxaliplatin desensitisation could only be performed in the main hospital setting and was not approved for use on the mobile chemotherapy unit.

### Grading of hypersensitivity reactions

The Common Terminology Criteria for Adverse Events, version 3.0 [24], were followed to grade the oxaliplatin-related HSRs both for the initial and breakthrough HSRs. Accordingly, grade 1 reactions were mild, with patients presenting only transient symptoms including flushing, rash, and a temperature below 38 °C. Grade 2 reactions were moderate and patients presented with symptoms such as rash, urticaria, dyspnoea, and a temperature > 38.0 °C. Grade 3 reactions were severe and patients presented with symptoms such as bronchospasm, angioedema/oedema and hypotension, or prolonged moderate symptoms that did not resolve. Grade 4 reactions were life-threatening and were characterised by anaphylaxis.

Back pain, which is a common symptom, was considered a Grade 1 reaction when it was present as the only symptom, according to our protocol.

### Outcome measures and statistical analysis

The primary objective of this study was to evaluate the safety and efficacy of oxaliplatin DDP at our hospital. The type and severity of initial and breakthrough HSRs were summarised using absolute numbers and relative frequency tables. Oncological outcomes were also assessed and presented. These included disease response to treatment and completion of the intended treatment cycles. For neoadjuvant treatment, this was defined as completion of planned chemotherapy cycles until a pre-planned imaging evaluation was performed. For adjuvant treatment, it was defined as completion of the pre-planned chemotherapy regimen. For palliative intent, it was defined as completion of planned treatment until either a subsequent scan or other clinical factors, such as unacceptable toxicity, indicated the need to stop treatment.

A secondary objective was to explore potential risk factors for BTRs, including age, sex, history of pre-existing allergies, treatment intent, previous exposure to oxaliplatin as part of an oxaliplatin-based regimen in prior lines of chemotherapy, chemotherapy regimen, grade of initial reaction, number of cycles in which the initial HSR occurred, and cumulative doses of oxaliplatin received at the time of the initial and BTR HSR.

To assess statistical differences between patients who experienced a BTR and those who did not, a chi-squared test was performed on all variables. Continuous variables were categorised based on clinical relevance and previous literature to facilitate comparison. The number and percentage of patients in each category were calculated and reported using the IBM SPSS Statistics, Version 31 program.

## Results

### Patient characteristics and initial oxaliplatin reaction

A total of 66 patients (36 females [55%]) with a median age of 60 years (range 36–81) were treated within the DDP. Most patients had colorectal (*n* = 35, 53%) or oesophago-gastric (*n* = 26, 39%) cancer, and the majority were treated with palliative intent (*n* = 56, 85%). Around half of the patients (*n* = 34, 52%) had oxaliplatin exposure in previous lines of chemotherapy, and 22 patients (33%) had a documented history of allergy in their electronic medical records (Table 1).

**Table 1:** Baseline demographics and characteristics of participants

	Number, *n* (%)
**Gender**	
Male Female	30 (45) 36 (55)
**Age**	
median (range)	60 (36–81)
**Type of Cancer**	
Colorectal Gastric Oesophageal Gastro-Oesophageal Pancreatic NET Appendiceal Cholangiocarcinoma	35 (53%) 11 (17%) 6 (9%) 9 (14%) 1 (2%) 1 (2%) 2 (3%) 1 (2%)
**Regime during initial hypersensitivity reaction**	
OxMdG/Rechallenge OxMdG OxMdG + Bevacizumab OxCap/Rechallenge OxCap OxCap + Trastuzumab OxCap + Nivolumab OxCap + Pembrolizumab FOLFIRINOX FLOT	26 (39%) 2 (2%) 31 (47%) 1 (2%) 1 (2%) 2 (3%) 2 (3%) 1 (2%)
**Treatment intention**	
Palliative Adjuvant Neo-Adjuvant	56 (85%) 8 (12%) 2 (3%)
**Oxaliplatin exposure in previous lines of chemotherapy**	
Yes No	34 (52%) 32 (48%)
**History of previous allergies**	
Yes No	22 (33%) 44 (67%)

The initial HSR was reported after a median of six cycles (range 1–26). The mean cumulative dose of oxaliplatin was 870 mg (range 130–2,295 mg). A grade 1 reaction was observed in 11 patients (17%), a grade 2 reaction in 48 patients (73%), and a grade 3 reaction in seven patients (11%). No patients experienced a grade 4 reaction. The most common symptoms were flushing/warmth (35%), dyspnoea (35%), oropharyngeal dysaesthesia/laryngospasm (24%) and pruritus (20%) (Table 2). One patient with oxaliplatin induced immune thrombocytopenia immediately following oxaliplatin infusion, which is considered a non-immediate HSR, was included in the study.

**Table 2:** Characteristics of initial and breakthrough reactions

	Oxaliplatin cycles prior to initial reaction median (range)	Desensitisation cycles - median (range)
	6 (1–26)	3 (1–19)
*Severity of Reaction*	Initial - Nr (%)	Breakthrough Nr (%)
Grade 1	11 (17)	7 (43.8)
Grade 2	48 (73)	7 (43.8)
Grade 3	7 (11)	2 (12.5)
Grade 4	0 (0)	0 (0)
*Type of Reaction*	Initial Nr (%)	Breakthrough Nr (%)
Pruritus	13 (20)	3(19)
Rash	10 (15)	4 (25)
Erythema	7 (11)	2 (13)
Urticaria	10 (15)	1 (6)
Flushing/Warmth	23 (35)	7 (44)
Dyspnoea	23 (35)	0 (0)
Oropharyngeal dysaesthesia/Laryngospasm	16 (24)	3 (19)
GI Symptoms (diarrhoea, nausea, vomiting)	11 (17)	3 (19)
Pyrexia	4 (6)	0 (0)
Rigors	2 (3)	0 (0)
Chest pain/Tachycardia	2 (3)	0 (0)
Angioedema	0 (0)	0 (0)
Back pain	4 (6)	2 (13)
Hypertension (SBP > 170mmHg)	3 (5)	2 (13)
Hypotension (SBP < 90mmHg)	4 (6)	0 (0)
Bronchospasm	1 (2)	1 (6)
Hypoxia (SaO2 < 94%)	2 (3)	0 (0)
Numbness/paraesthesia/weakness	6 (9)	0 (0)
Miscellaneous neurological symptoms (visual blurriness/slurred speech/imbalance/dizziness)	7 (11)	0 (0)

### Breakthrough reactions

The total success rate was 76% and only sixteen patients (24%) experienced a BTR: 69% in the first cycle of DDP treatment and 31% in cycles two to six. The median number of oxaliplatin cycles on the DDP regimen was three (range 1–19), with a mean cumulative dose of 673 mg (range 90–5602 mg). Most of the reactions were grade 1 or 2 (87.5%). Only two patients experienced a grade 3 reaction. The most common BTRs were flushing/warmth (*n* = 7/16, 44%), rash (*n* = 4/16, 25%), pruritus (*n* = 3/16, 19%), oropharyngeal dysaesthesia (*n* = 3/16, 19%), and gastrointestinal symptoms (e.g. diarrhoea, nausea, vomiting) (*n* = 3/16, 19%) (Table 2). No life-threatening or fatal reactions were reported in patients who received the DDP.

Our protocol dictated that, once a patient experienced a BTR, oxaliplatin treatment should be permanently discontinued, regardless of the nature of the reaction. However, two patients continued their treatment with the DDP despite experiencing a BTR. In one case, oxaliplatin was continued with a 25% dose reduction at the final dilution—where the BTR had occurred—and with an additional dose of chlorphenamine and hydrocortisone administered beforehand. In the other case, treatment proceeded without any further modifications. Neither patient experienced additional HSRs.

### Risk factors for breakthrough reactions

The potential risk factors related to BTRs were analysed, including age, gender, type of cancer, allergy history, cumulative dose, curative or palliative intent, chemotherapy regimen, use of targeted agents, and oxaliplatin exposure in previous lines of chemotherapy; however, no statistically significant results were found for any of the studied parameters (Table 3). In patients with BTR, there was a trend of receiving fewer than three cycles of treatment (*p* = 0.07). Patients who did not develop HSRs received a higher median cumulative dose of oxaliplatin than those who reacted while on DDP (*p* = 0.01).

**Table 3:** Risk factors for breakthrough reactions

Risk factors	Group Categories	Chi-square (x2) test	*p*-Value
Age	< 65/≥65	0.102	0.750
Gender	Male/Female	3.664	0.160
Type of Cancer	UGI/LGI/HPB	0.748	0.668
Treatment intention	Curative/palliative	1.302	0.254
Regime before the reaction	CAPOX/FOLFOX/ FOLFIRINOX/FLOT	3.241	0.663
Targeted agents	Panitumumab/Bevacizumab /Nivolumab/Trastuzumab	0.206	0.650
Previous Exposure to oxaliplatin	Yes/No	0.019	0.889
Initial Grading of the reaction	Mild-moderate/Severe	0.003	0.957
Hx of Allergy	Yes/No	2.640	0.104
No of cycles in desensitisation protocol	3 or less/>3	3.284	0.070
Cumulative dose at the time of initial reaction	< 500/500–1000/>1000	1.142	0.565
Cumulative dose at the time of end of treatment	< 500/500–1000/>1000	9.300	0.010

### Oncologic outcomes

Of the 66 patients in total, 34 achieved a favourable outcome in the initial scan after DDP was introduced. Of these patients, 25 were in a palliative care setting, two were receiving treatment in a neoadjuvant setting, and seven were undergoing adjuvant treatment.

**Fig. 2:**
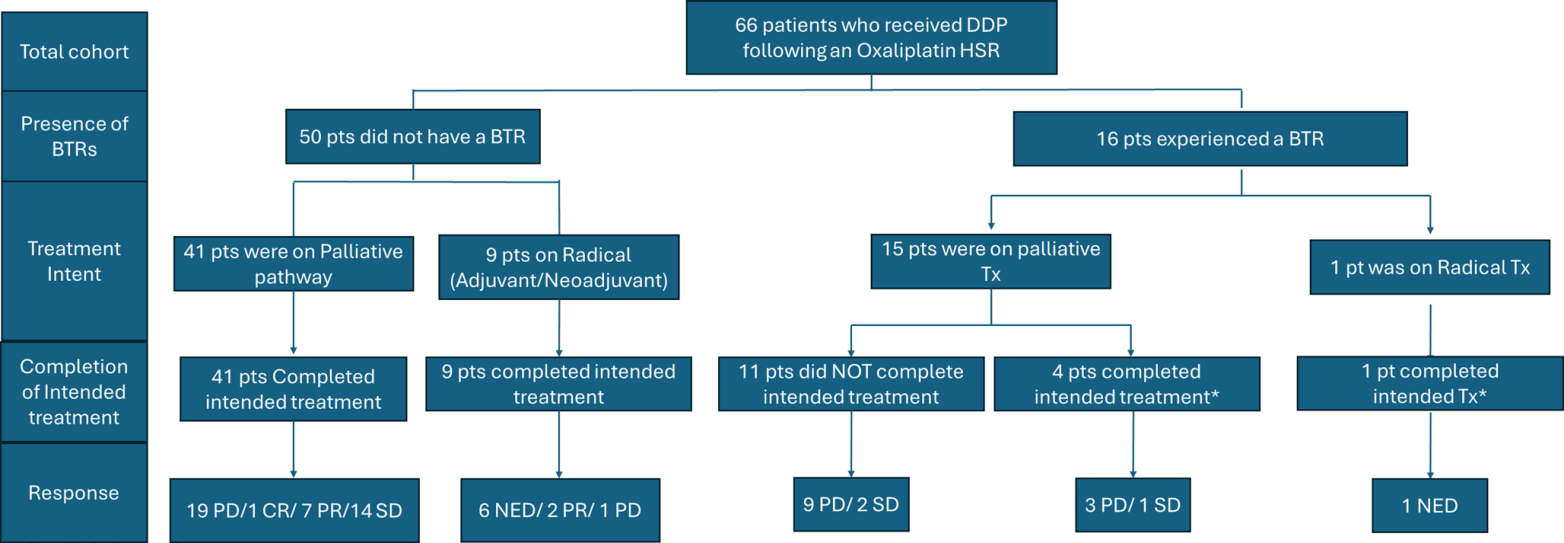
Oncological outcomes. *T*his figure illustrates the oncological outcomes of patients categorised by the presence or absence of BTR, treatment intent, and completion of treatment. BTR, Breakthrough Reactions, DDP Drug-Desensitisation Protocol, Pts: patients, PD: Progression of Disease, CR: Complete Response, PR: Partial Response, SD: Stable Disease, NED: No Evidence of Disease. Include patients who completed their intended treatment despite having undergone BTR

Fifty-five patients (83%) completed the intended number of oxaliplatin cycles, indicating that the initial reaction did not require treatment to be stopped prematurely and switched to a different chemotherapy regime (Fig. 2).

Fifty of the patients who completed their intended treatment did not experience a BTR. Nine were treated with curative intent, two were on neoadjuvant treatment and showed some response, and sever were on adjuvant treatment. Out of those six showed no evidence of disease and one, with colorectal cancer, experienced disease recurrence. The remaining 41 patients were on palliative chemotherapy. Of these, 19 showed progressive disease (PD) on the first assessment scan after the introduction of the DDP, 14 achieved stable disease (SD), seven had a partial response (PR) and one attained a complete response (CR).

Five patients who completed the intended therapy had a BTR. Four of these patients were receiving palliative treatment. Two stopped treatment due to disease progression, while the other two continued at the discretion of their oncology team: one experienced disease progression, while the other achieved stable disease (SD). The final patient was on adjuvant therapy and treatment was stopped due to other treatment-related side effects, with no evidence of recurrence.

## Discussion

Over the past two decades, oxaliplatin-related HSRs have been the subject of thorough investigation, resulting in the development of various DDP aimed at mitigating these events. Despite the publication of over 30 studies on this subject, a consensus remains elusive, and practices vary among centres according to local policies.

Prominent studies in this field [13,16,27–30], have shown that pre-medication regimens in desensitisation protocols typically consist of three main components: antihistamines, corticosteroids and additional agents tailored to the characteristics of the initial HSR. As oxaliplatin-related HSRs are primarily considered to be type I IgE-mediated reactions, with histamine as a key mediator, antihistamines are a consistent feature across all protocols. These generally include both H1 and H2 receptor antagonists, although the specific timing and dosage can vary. A review of H2 antagonists used for premedication in paclitaxel chemotherapy found no benefit in reducing HSRs, which calls into question their effectiveness in preventing oxaliplatin-induced HSRs [31,32].

Although corticosteroids are widely used to treat various inflammatory conditions, ranging from sepsis to immunotherapy-related reactions, they are not included in all desensitisation protocols. Similar to previous oncology-led approaches, our protocol incorporates the administration of high-dose of dexamethasone prior to oxaliplatin [27,30]. On the other hand, allergist-led protocols usually don’t include corticosteroids unless the initial reaction included symptoms like chills, shivering, fever or pain. In such cases, additional agents such as intravenous fluids, ibuprofen and opioids are commonly used [13,28]. Furthermore, some protocols include aspirin and montelukast as part of the pre-medication regimen for patients who experienced flushing or bronchospasm during their initial HSR [13,16] - an approach not adopted in our protocol.

The original protocol for oxaliplatin desensitisation at Brigham and Women’s Hospital involved three different dilutions administered in 12 steps over a 6-hour period [18]. However, various teams have introduced modifications to this protocol, either increasing the number of steps and dilutions [16,29] or modifying the protocol depending on the type and severity of the initial and breakthrough HSRs [13,28]. Due to the complexity of the original protocol, simplified versions using fewer dilutions and infusion steps have also been developed and studied [27,30], including our own, which has been shown to be equally effective.

In our study, we observed a total success rate of 76%, which is similar to that reported in previous studies, irrespective of whether multi-step or simpler graded protocols were employed [13,16,27–30]. This suggests that the success of the DDP may depend on other underlying factors, in addition to the number of steps or adjustments to pre-medication. A recent study proposed that 26.0% of patients with oxaliplatin-induced adverse drug reactions (ADRs) could not undergo oxaliplatin rechallenge. A specific HLA-DRB*12:01 allele has been identified as conferring sensitivity to these reactions, which could explain the consistent outcome observed across the studies [33].

Only two patients (3% of the population) experienced a grade 3 HSR, and there were no grade 4 or 5 (fatal) HSRs. This is consistent with similar studies who reported 5%^31^ −7%^28^ of severe grade 3 and 4 reactions. The overwhelming majority of BTRs were classified as Type I HSR. No type II reactions involving antibody-mediated immune reactions in which IgG or IgM antibodies bind to antigens on the surface of cells or extracellular matrix, leading to cell destruction, inflammation, or dysfunction. were observed, and none of the patient’s required hospitalisation.

The management of BTRs during DDPs remains challenging. While permanent discontinuation of oxaliplatin is recommended by our protocol, several strategies have been explored to mitigate their occurrence. Other approaches involving simplified DDPs included switching to a 12-step desensitisation protocol, which was only partially effective, as two-thirds of patients still experienced further reactions and had to discontinue treatment [30]. More recent reports have also explored the efficacy of new immunomodulating agents such as tocilizumab [34], omalizumab [28,35]or acalabrutinib [36], as an attempt to avoid BTRs with promising results. However, larger studies are needed to confirm the effectiveness of these methods.

It is challenging to identify patients predisposed to BTRs due to the absence of reliable, consistent outcome data. Madrigal-Burgaleta et al [37]. found that atopy was a risk factor for BTRs in a mixed cohort of patients receiving taxanes, platinum compounds, or biological agents (OR: 2.16; 95% CI: 1.5–14.06; *p* = 0.03). However, previous studies have not identified age, gender or atopy as risk factors for BTRs [13,38,39], which is consistent with our results.

Caiado et al. showed that receiving more than ten platinum infusions was a risk factor for BTRs (OR: 4.11, 95% CI: [1.17–14.52]; *p* = 0.03), and this was confirmed as an independent risk factor in a multivariate analysis [39]. However, our data indicate that the cumulative dose of oxaliplatin was not a significant risk factor [35]. This difference may be due to the fact that their population included patients who had also received other platinum treatments, such as cisplatin or carboplatin [28]. Caiado et al. also so demonstrated that an initial HSR of grade 2 or 3 increased the risk of BTRs (OR: 2.19, 95% CI: [0.38–12.79]), though this difference was not statistically significant [39]. Similarly, we found no difference in the incidence of BTRs between patients with mild/moderate and severe initial HSR. Silver et al. suggested that patients with BTRs tend to do so within the first three cycles of DDP [13], and our data did reflect the same trend.

Efforts have been made to study blood biomarkers and skin testing (ST) as predictors of response to DDP. Although serum tryptase is generally considered a reliable marker of anaphylaxis, research has shown that it is not useful in predicting BTRs [13]. IL-6 levels are significantly raised in patients presenting with cytokine release syndrome [13]. An increased total IgE level (> 100 U/mL) was shown to be a risk factor for BTRs, (OR: 8.24, 95% CI: [2.06–30.02]) but this study involved a mixed population who received different types of platinum agents [39]. Studies regarding the ST protocol are controversial. Some studies have shown that positive oxaliplatin skin testing can predict a response to DDP [16,38], and a stratification protocol has been implemented for the use of DDP [29], suggesting positive and negative predictive values of 92% and 56.4%, respectively. However, other studies have not confirmed these outcomes [13,39]. Unfortunately, such parameters were unavailable and therefore not included in our study.

We categorised both the initial and the breakthrough reactions based on the clinical presentation and severity of the reaction, according to the CTCAE criteria for allergic reactions/hypersensitivity (version 3.0) [25]. However, attempts have been made to endophenotype oxaliplatin HSRs according to their underlying pathophysiology, separating type II reactions (pruritus, urticaria, angioedema, nasal congestion, sneezing, wheezing, coughing, throat tightness, tongue swelling and hypotension), which are IgE-mediated reactions related to increased tryptase levels, from cytokine release reactions (chest pain, back pain, headache, other pain, chills, rigours, fever, numbness/weakness and hypertension/hypotension), which are characterised by increased IL-6 levels, as well as HSRs that can be mediated by either of these pathophysiological mechanisms [13]. This classification not only allows adjustments to the initial DDP depending on the type of reaction, but also modifications to the DDP if a patient experiences a HSR [13,28].

While the primary objective of DDP is to enable treatment to continue in patients with HSRs, it is ultimately crucial for maintaining favourable oncological outcomes. Previous studies have shown that desensitisation protocols for various chemotherapy agents can achieve similar oncological outcomes to standard, non-desensitised regimens [40,41]. Nevertheless, large-scale data remains scarce.

The largest study of oxaliplatin DDP to date was conducted by Alonso Martinez et al., who reported a PD rate of 35% [30] . In our cohort, 83% of patients completed their intended treatment; however, 44% experienced disease progression. Eighteen per cent of patients discontinued treatment due to other toxicities, while 44% completed their full course. Interpreting progression rates in these settings is complex owing to the heterogeneity of patient populations, which often include individuals undergoing both radical and palliative treatments, as well as those with different primary diseases. Further research involving larger, more homogeneous patient groups is needed to establish whether oxaliplatin-related HSRs or the nature of the desensitisation protocol itself may have a negative impact on oncological outcomes.

Our study has several limitations. Data on initial and breakthrough HSRs were collected retrospectively from electronic medical records at a single centre. Consequently, the characterisation and grading of reactions depended heavily on the quality and completeness of clinical documentation, which may have varied depending on the experience of the healthcare professional involved. The cohort predominantly included patients with Grade 1–2 reactions, as our local policy excludes those with moderate-to-severe initial reactions from desensitisation. Additionally, as the study reflects the experience of a single centre using a desensitisation protocol that has not yet been adopted by other teams, the findings may be difficult to generalise. The small sample size also precluded a multivariate analysis to explore potential risk factors for breakthrough reactions. Despite these limitations, our results suggest that the DDP is a reliable treatment for patients with oxaliplatin reactions, provided the appropriate patients are selected.

## Conclusion

To our knowledge, this is one of the largest published series of patients treated with oxaliplatin using a structured desensitisation protocol, which further supports the safety and clinical efficacy of this approach in managing oxaliplatin-induced HSRs. The high overall success rate highlights the value of desensitisation in enabling patients to continue with life-prolonging chemotherapy, even following a significant hypersensitivity event.

However, some patients will still experience BTRs or be unable to tolerate re-exposure despite desensitisation. There is still a need to establish standardised, consensus-driven DDPs across institutions. This would facilitate multi-centre collaborations, increase statistical power and enable more rigorous exploration of predictive biomarkers and clinical risk factors associated with DDP failure.

Crucially, identifying patients at high risk of desensitisation failure early on could prompt timely referral to specialist allergy services. In these settings, expert allergists can implement tailored interventions, such as stepwise desensitisation and adjunctive pharmacological premedication, thereby maximising the likelihood of treatment continuity and oncological benefit.

In conclusion, although DDPs are a valuable tool for overcoming oxaliplatin HSRs, the future lies in improving patient selection, personalising management and encouraging collaboration to optimise outcomes in different clinical settings.

## Data Availability

The datasets used and/or analysed during the current study are available from the corresponding author on reasonable request.
